# Same-different learning of odour stimuli in dogs

**DOI:** 10.1007/s10071-025-02035-z

**Published:** 2026-01-18

**Authors:** Claire Ricci-Bonot, Amelia Duncan, Daniel S. Mills, Thomas W. Pike, Helen Zulch, Victoria F. Ratcliffe, Michael Nickson, Emma Hobbs, Anna Wilkinson

**Affiliations:** 1 Animal Behaviour, Cognition and Welfare Group, Dept. of Life Sciences, University of Lincoln, Lincolnshire, LN6 7DL UK; 2 Defence Science and Technology Laboratory, Porton Down, Salisbury, UK

**Keywords:** Canine, Cognition, Concept learning, Odour detection, Same-different, Odour space

## Abstract

The performance of detection dogs relies on their ability to detect and alert to variations of the stimuli upon which they have been trained. As such, research has tended to focus on understanding the likelihood of generalising beyond a trained stimulus set. However, it remains unclear which stimuli dogs perceive as the ‘same’ or ‘different’ to others. Understanding this perception would allow the creation of appropriate training aids to improve the performance of working dogs. The aim of this study was to establish whether dogs were capable of same-different concept learning with odours and whether they could generalise this learning to a novel stimulus set. Dogs were presented with two odours simultaneously and trained to give one indication behaviour if the samples were the same, and an alternative indication if they were different. Four of the ten dogs tested were able to meet the learning criteria, indicating that they could learn the task with the training stimuli. However, none were able to generalise the concept to a new stimulus set. The failure of the dogs to generalise the same-different learning to novel stimuli suggests that the procedure used, while showing some promise, may not be the best approach to assess how dogs perceive odours in relation to each other.

**Supplementary Information:**

The online version contains supplementary material available at 10.1007/s10071-025-02035-z.

## Introduction

Scent detection dogs are used in a variety of situations including to search for drugs (Aviles-Rosa et al. 2021), explosives (Furton and Myers 2001), missing people (Greatbatch et al. 2015), cadavers (Lasseter et al. 2003), diseases (e.g. cancer: McCulloch et al. 2006; Horvath et al. 2013; reviewed by Moser and McCulloch 2010), and in wildlife conservation (Cristescu et al. 2015; and reviewed by Beebe et al. 2016). They are typically trained using either real samples of the target odour(s) of interest, or with synthetic training aids (Moser et al. 2019). However, the odour profile of target odours can vary greatly depending on how they are sourced, manufactured and handled (Cerna et al. 2011; Oxley and Waggoner 2009) and so developing effective training aids which allow appropriate generalisation to the range of target odours experienced operationally is challenging. Thus, in order to optimise training aid development, it is essential to understand when dogs perceive odours as similar to each other or different from each other.

The likelihood of dogs responding in similar ways to different odours depends on generalisation and discrimination. Discrimination is the ability of an individual to respond differently to stimuli which are perceived as different (Guttman and Kalish 1956; Pearce 1994). In contrast, generalisation is the ability of an individual to respond in the same way to stimuli which are perceived as similar because they share common features, for example in their physiochemical properties (Stokes and Baer 1977; Thompson 1995; Ghirlanda and Enquist 2003). Generalisation is usually illustrated as a Gaussian curve representing sensitivity to variations of a trained stimulus; an increase in dissimilarity between the trained and the trained target (exemplar) stimulus results in a decrease in the probability of responding. When the curve is broad this indicates greater stimulus generalisation, whereas a narrow curve indicates greater discrimination (Ghirlanda and Enquist 2003). This concept is used to explain how dogs’ perception of similarity between odours is affected by the properties of these stimuli (Caldicott et al. 2024). Differences in carbon chain lengths or functional groups influence the likelihood of an animal generalising. For example, both dogs and rats have been shown to generalise between two chemicals with similar carbon chain lengths if they differed by only one carbon atom regardless of whether they were alcohols or carboxylic acids (Cleland et al. 2002; Hall et al. 2016). However, generalisation performance gradually deteriorates as the carbon chain length increases (Cleland et al. 2002; Hall et al. 2016). By contrast, in another study, dogs struggled to generalise between stimuli with similar carbon chain lengths when they varied in their functional groups (Simon et al. 2018). The limitation of this sort of approach is that it may not lend itself well to optimizing training and developing training aids. Approaches that emphasize the relations between training stimuli may be more appropriate in this context.

One potential approach to optimising training aid selection is to use same-different concept learning, where individuals classify unknown stimuli on the basis of whether or not they are perceived to be the same as or different from another stimulus presented at the same time (Katz et al. 2007; Katz and Wright 2021). From this approach the perceptual space can be constructed to incorporate information regarding the relative relationship between the stimuli (Katz et al. 2007; Katz and Wright 2021). From this it would be possible to predict which odours can be perceived as the same or different from each other and to develop training aids that leverage this knowledge in order to promote the widest detection capacity in trained dogs.

Same-different concept learning in dogs has been investigated in relation to visual stimuli (Tapp et al. 2004; Gadzichowski et al. 2016; Byosiere et al. 2017; Scagel and Mercado 2023), but, to our knowledge, has not previously been assessed using odour stimuli. However, there is evidence that dogs can learn to match a sample stimulus to the same stimulus presented in an array of different stimuli (Lazarowski et al. 2021); this ability suggests that they may have the capability to learn a same-different concept. In this latter study, after smelling the sample stimulus, dogs were required to investigate 6 different pots containing odours which were placed in an arc, and had to indicate the one containing the target odour by sitting. Of these 6 stimuli, only one contained the target odour, the others were either empty (*n* = 4) or contained a different odour (*n* = 1). Dogs were able to learn the task and transfer this learning to novel stimuli. In another study, dogs were trained to match a Body Scent (scent collected from direct handling) or Trace Scent (scent from clothing or objects) odour to the same scent in an array of different Body Scent or Trace Scent stimuli (Marchal et al. 2016). In this study, dogs were trained to match a Trace Scent collected from a crime scene with either the Trace Scent or Body Scent from the suspects/victims, for use in judicial cases. Dogs were trained to smell a target odour and then inspect the line-up of stimuli (containing both Trace Scents and Body Scents; *n* = 5) and lie down beside the scent that matched the target odour. The dogs managed to correctly identify the correct target odours in around 85% of cases and never alerted to non-target human odours, suggesting they were able to match the samples and transfer between the two different odour collection methods.

Matching to sample tasks allow us to assess which stimulus an animal perceives as most similar to a sample stimulus, however, they do not allow us to determine how similar or different the dogs perceive the stimuli themselves to be (Johnen et al. 2017). Therefore, in the current study, we used the novel approach of same-different concept learning to assess perception of odours in dogs, by adapting the method developed by Nakayama et al. (2022) to assess perceptual distances between odours in mice. Our task presented two odours simultaneously for the dogs to smell, after which they indicated whether the stimuli were perceived as the same or different by responding in one of two trained ways. For example, if they were perceived as the same, the dog would respond by sitting, while if they were perceived as different, it would respond by lying down. An important feature of this method is that dogs should base their decision on the comparison of similarities between the two stimuli rather than by eliminating stimuli that are too different from the target odour (Johnen et al. 2017).

The aim of this study was therefore to establish whether dogs were capable of learning a same-different task with odours and whether they could transfer this learning to novel odours.

## Materials and methods

The study was conducted at the University of Lincoln (Lincoln, United Kingdom), at Minster House (Brayford campus) and at the Rural Science Centre (Riseholme Campus) between May 2023 and October 2024. Dogs were recruited via the ‘PetsCanDo’ of volunteer dog owners at the University of Lincoln. In total we recruited 16 dogs.

### Animal subject selection

On a first visit, potential dogs for consideration were brought individually into the testing room where they were allowed to freely investigate off-lead to become accustomed to the environment and the experimental set up. This habituation period finished once the dog sat, lay down or returned to the experimenter in a relaxed or positively expectant manner. The maximum time allowable for habituation was set at 20 min. If the dog did not habituate within this time they were excluded from the study. Subsequently, all animals underwent a second screening process in which they had to learn to respond to odour stimuli (essential oils) using one of two different behaviours depending on whether the odours presented were the same or different (Supplementary information ‘Pre-training’ and Table S1). These behaviours (e.g. sit, lie down, chin rest, paw on a box, go to a mat) were chosen depending on individual dog or owner preference (e.g. if the dog had musculoskeletal difficulties, then sit/lie down was avoided). Where possible, responses were balanced between dogs. Of the 16 dogs initially recruited, 10 dogs of various breeds, aged 1–8 years old (mean ± SD: 4.70 years, ± 2.21), including 4 males and 6 females, proceeded to formal training for the task (Table 1).

**Table 1:** Subject dogs’ characteristics and information (Note: * = dogs having previously taken part in scent experiment and/or scent training/competition)

Dog	Breed	Age (years)	Gender	Behaviour for ‘Same’	Behaviour for ‘Different’
Wren*	Golden Retriever	8	Female	Down	Sit
Fable	Pomsky (Pomeranian x Husky)	1	Female (neutered)	Mat	Chin
Alfie	Cockerpoo	6	Male (neutered)	Paw	Mat
Freda	Labrador x Golden retriever	6	Female (neutered)	Mat	Chin
Hector*	Nova Scotia Duck Tolling Retriever	7	Male (neutered)	Sit	Down
Ghillie*	Cocker Spaniel (Working line)	5	Male (neutered)	Chin	Mat
Sol*	Cocker Spaniel (Working line)	2	Male	Down	Sit
Talisker	Cocker Spaniel (Working line)	3	Female	Down	Sit
Sumi	Labrador x Golden retriever	4	Female (neutered)	Paw	Chin
Connie	Labrador Retriever	5	Female (neutered)	Chin	Mat

### Experimental set-up

**Fig. 1:**
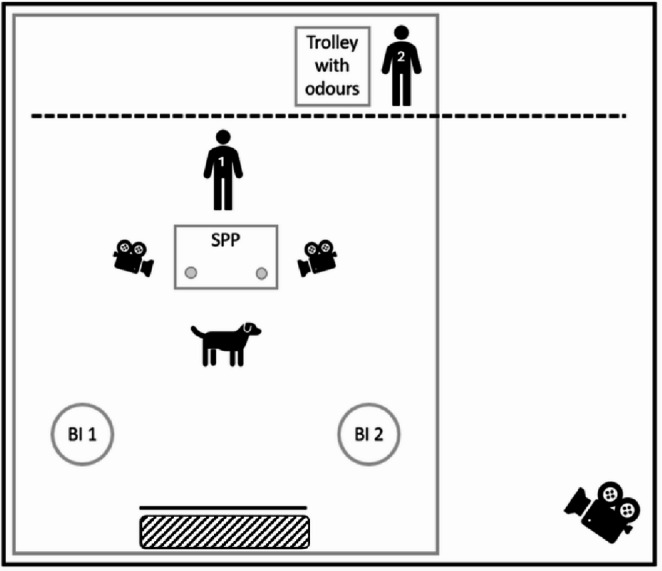
Diagram showing the experimental set-up, showing the location of the two experimenters and dog. The black dashed line shows the location of the panel. The stimulus presentation platform (SPP), on which there are two scent pots (with a diameter of 60 mm and a space between of 320 mm) containing the glass vials (measuring 19 × 65 mm) which contain the odour stimuli in liquid or solid form, is placed centrally to Experimenter 1 and the two behaviour items (BI; may include a chair for chin rest, a small box for paw or a mat; these are not present for dogs with sit and down as their indication behaviours). The camera icons show the location and direction of cameras used during trials. The striped area shows the area where the food rewards are given during the test trials (located behind a screen, denoted by the bold black line)

Dogs were trained and tested in an experimental room. A diagram of the experimental setup is shown in Fig. 1. Dogs had free access to water throughout training.

### Stimuli

In the training phase, dogs were trained on a set of 10 odours consisting of common household items; these were selected because they had already been successfully used in previous odour detection research (Supplementary information Table S2) and differ substantially from each other (at least to human perception). Each of these odours were made up at two different concentrations (one low, i.e. just about detectable by humans; weak odour and one high, i.e. very detectable to humans; strong odour), resulting in 20 different stimuli, with the following possible combinations:

- 20 identical combinations of the same odour at the same relative concentration (e.g. S1 = paprika ‘high’ and S2 = paprika ‘high’).
- 90 combinations of different odours at the same relative concentrations (e.g. S1 = paprika ‘high’ and S2 = oregano ‘high’).
- 90 combinations of different odours at different relative concentrations (e.g. S1 = paprika ‘high’ and S2 = oregano ‘low’).

Each presentation pot contained a sample that equated to the predetermined stimulus volume for high or low odour concentration (Supplementary information Table S3).

The stimulus set consisted of 10 odours each at two concentrations (20 stimuli). Four of these stimuli (two odours at two concentrations) only appeared on what we call ‘same’ trials, these incorporate what will be referred to as ‘same only’ pairings. ‘Same’ trials consisted of one of the two odours being presented as a pair, at the same concentration within a trial (i.e., either both at high concentration or both at low concentration). Four of the stimuli (two odours at two concentrations) only appeared as a ‘different’ trial (i.e., one odour at high or low concentration paired with a different odour at high or low concentration), these incorporate what will be referred to as ‘different only’ pairings. The remaining 12 stimuli appeared in both ‘same’ or ‘different’ trial forms, these incorporate what will be referred to as ‘combined’ stimulus pairings. For example, the allocation of training stimuli for one dog might have been: pine essence and paprika only appeared on ‘same only’ trials, antibiotic cream and coffee only appeared on ‘different only’ trials and the remaining stimuli were ‘combined’ pairings and appeared in both ‘same’ trials and ‘different’ trials (e.g., pine essence was only ever paired with pine essence, and coffee was never paired with coffee). This allocation was different for each dog and counterbalanced across animals.

### Training procedure

At the beginning of each training session, dogs received four trials with the stimuli used in the screening process where the dogs first smelled the two target odours; Experimenter 1 then verbally cued one of the two behaviours (depending on whether the stimuli were ‘same’ or ‘different’). This procedure was used to ensure continuity even after several days without training.

When presented with the training stimuli, dogs were given the verbal cue “go sniff”; they did not then receive any verbal cues for a specific behavioural response after sniffing both samples. Experimenter 1, who presented the pots, was blind to the correct response, and Experimenter 2 verbally indicated if the response was correct or incorrect; Experimenter 1 then provided verbal praise accompanied by a food reward if correct. Experimenter 2 was hidden behind a screen and so could not be inadvertently cueing the dog. Each dog received blocks of 10 trials and each block was always made up of five ‘same’ and five ‘different’ trials. They were pseudo-randomly presented so that there were no more than two ‘same’ or two ‘different’ trials consecutively. During the training phase, dogs typically carried out up to 2 blocks each day, however, we found that some dogs remained motivated to work for up to 5 blocks in a single day. Each block typically lasted up to 15 min, and dogs who were unable to remain focused for a block would be given a break when required.

The correct behavioural response was reinforced with food and verbal praise whereas incorrect responses resulted in a time out before a repeat of the trial was presented. The length of the time outs varied depending on the dog, varying from 5 to 30 s. If the dog responded incorrectly to the odour stimuli on the second trial attempt, Experimenter 1 gave a verbal cue for the correct indication behaviour once the dog had sniffed in all further repeats of the trial, until the dog gave the correct indication behaviour.

The performance of dogs in each block was assessed on the basis of the number of correct indications.

Once dogs reached the criterion of an average of 7 out of 10 correct responses (for ‘same’ and ‘different’) over 3 blocks (cumulative binomial probability estimate for at least 21/30: *p* = 0.0214), with the added constraint that no block had 5, or fewer, out of 10 correct responses, they were considered to have learned the task and progressed to the testing phase.

### Testing

Pilot testing with three of the dogs revealed that running test trials using standard procedures (e.g. Wright et al. 2017), which included no differential reinforcement on the test trial, prior to moving on to the next training trial, resulted in an extinction of the learned response. Therefore, in the following testing phase, each test stimulus combination was only presented once, in order to allow positive reinforcement of the responses using food; all responses received this reinforcement regardless of whether the dogs’ indication behaviour was considered correct. This approach was used to reduce the likelihood of learning whilst also keeping the dogs motivated (e.g. Mueller-Paul et al. 2014). The three dogs that took part in pilot testing returned to the training phase and needed to reach criteria again before moving to the main testing phase. One dog from this cohort did not achieve the learning criteria again and so did not move on to the main testing phase (Supplementary information Table S6).

### Test stimuli

Test stimuli were short carbon chain molecules which differed by carbon chain length or functional group. These have previously been used in olfaction research in dogs and their standard generalisation curves are known (Hall et al. 2016; Simon et al. 2018) (Table 2, Supplementary information Table S4 and Table S5). Esters were added to the stimulus set and used as a control, i.e. we know that they are almost certainly perceived as different from the other stimuli used (as they smell very different to humans) and consequently the dog was expected to respond ‘different’ when perceiving these even if they responded ‘same’ to all other stimuli. The combinations of test stimuli were selected based on their chemical structure/similarity as well as dogs’ generalisation data from published studies (Hall et al. 2016; Simon et al. 2018). All test stimuli were novel to the dogs.

**Table 2:** Stimuli selected for testing

Stimuli selected for the carbon chain length part	Stimuli selected for the functional group part	Esters which can be used in the two parts
Ethanol (2 C)	Pentanoic acid	Butyl butyrate
Propanol (3 C)	2 - pentanone	Ethyl cyanoacrylate
Butanol (4 C)	3 - pentanone	
Pentanol (5 C)	Pentanal	
Hexanol (6 C)	Methyl valerate (Methyl pentanoate)	
Heptanol (7 C)		
Octanol (8 C)		

### Test procedure

During the testing phase, the dogs received 12 trials in each test block: 10 training trials and 2 test trials (one was a same stimulus pairing and the other was a different stimulus pairing). These test trials were pseudo-randomly intermixed with training trials, presented between Trials 2 and 11, and were separated by at least one training trial (Wright et al. 2017). During the testing phase, dogs usually only carried out 1 block each day, however occasionally they were able to carry out 2 blocks in a day.

The test trials were identical to training trials except that Experimenter 2 indicated verbally ‘test’ after the dog had performed its behaviour. Experimenter 1 then said ‘thank you’, this response was followed by a low value reward in a location away from the main experimental area (opposite the stimulus presentation platform; Fig. 1) no matter which indication behaviour the dog performed. To reduce possible carryover of learning effects each stimulus pairing was only presented once to each dog.

Dogs needed to obtain a score of at least 70% in the training trials within a test block for it to be considered as meeting the criteria and for the test data to be used.

### Analysis

For the purposes of assessment, behavioural responses of the dogs during training were scored as correct when dogs gave a ‘same’ indication for ‘same’ samples, or a ‘different’ indication for ‘different’ samples. However, we were unable to assess whether test data on different samples were correct as we did not know which the dogs perceived as the same or different. Thus only the performance of the dogs when presented with ‘same’ combinations was assessed. We used binomial probability tests (P[X ≥ number of success]) to assess whether performance was greater than chance.

## Results

### Training

**Fig. 2:**
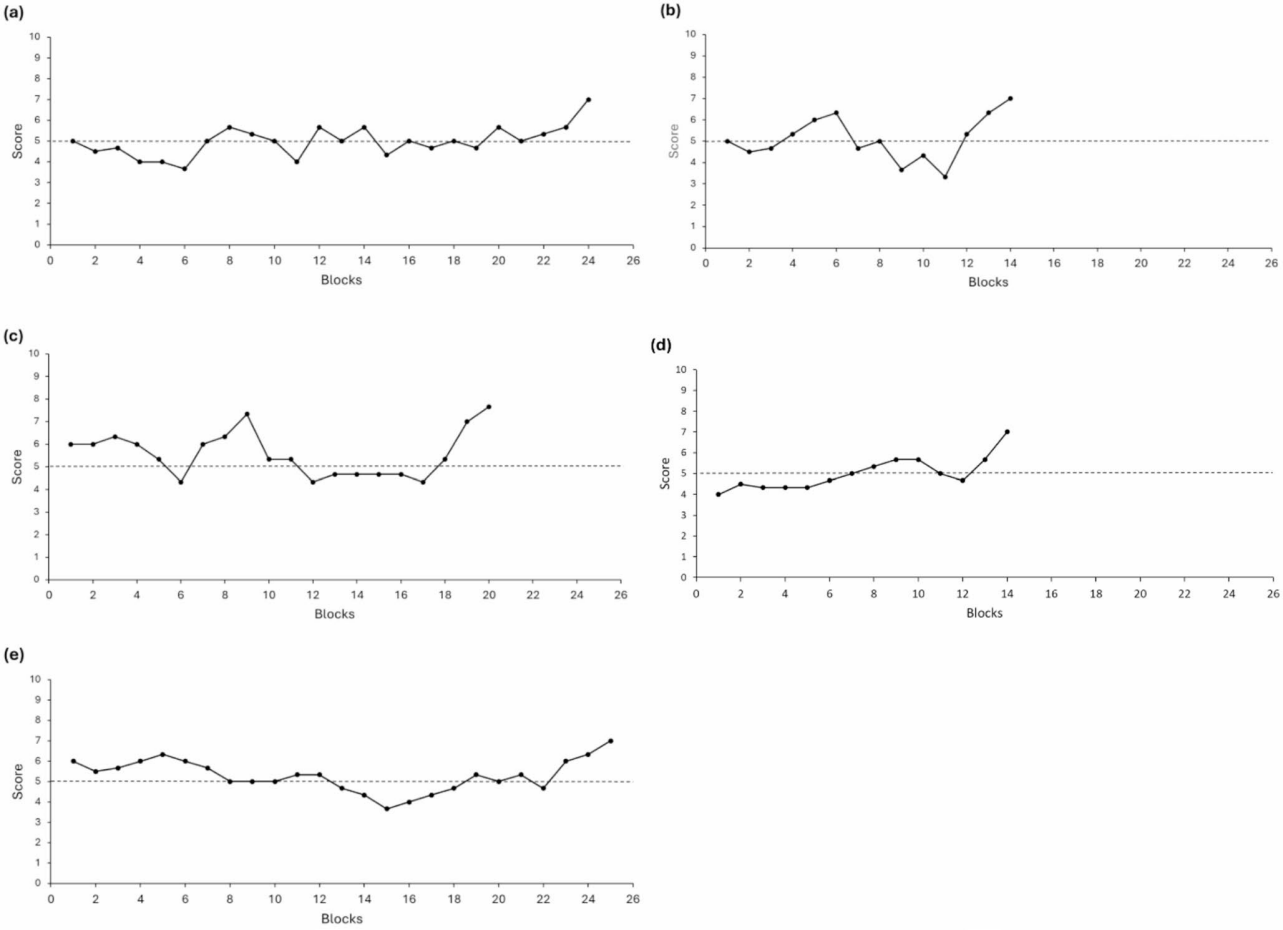
Training graphs for all the dogs who met the criteria in the training phase, showing number of correct responses during the training trials in each block for Wren (**a**), Fable (b), Alfie (**c**), Freda (**d**) and Hector (**e**). Chance levels of performance are denoted by the dashed horizontal line

5 dogs met the criteria and moved to the testing phase. They needed a mean of approximately 19 blocks, of 10 training trials each, to move from training to testing (Fig. 2, Supplementary information Table S6 and Figure S2). The fastest dog needed just 14 blocks, while the slowest took 25 blocks. Performance did not differ between stimuli that were only presented as the same and different compared to those presented as both (Supplementary information Figure S3).

5 dogs were removed from the experiment at this stage as they did not show progress in the training phase (Supplementary information Table S6). 1 further dog (Hector) was removed from the study following pilot testing so did not move on to the main testing phase.

### Testing

The fastest dog needed just 14 blocks, while the slowest took 20 blocks to complete all the test combinations.

Test trials revealed no evidence that the dogs preferentially chose ‘same’ (carbon chain length: *p* = 0.9961; functional group: *p* = 0.5000; Tables 3 and 4) on trials where there was no molecular difference between the stimuli. Consequently, we cannot draw general conclusions from the dogs’ performance whether the combinations presented were in fact different.

**Table 3:**
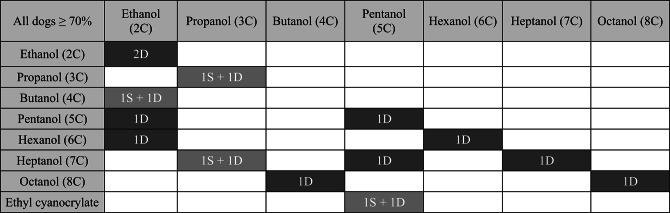
Perception as ‘same’ or ‘different’ of molecules which differ by carbon chain length (Note: Highlighted in black = dog(s) perceived them as different; highlighted in dark grey = answers from multiple dogs which differ from each other). The numbers indicate the number of dogs that encountered this combination and obtained a score ≥ 70% in the training trials for a block)

**Table 4:**
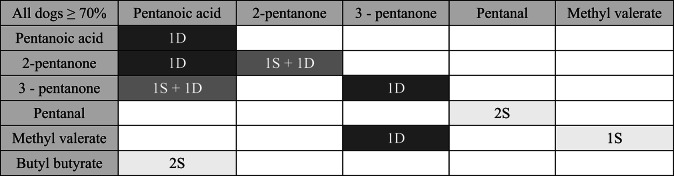
Perception as ‘same’ or ‘different’ of molecules which differ by functional group (Note: Highlighted in black = dog(s) perceived them as different; highlighted in light grey = dog(s) perceived them as same; highlighted in dark grey = answers from multiple dogs which differ from each other. The numbers indicate the number of dogs which encountered this combination and obtained a score ≥ 70% in the training trials for a block)

When looking at the overall performance, irrelevant of performance in the intermixed training trials, there is still no evidence that dogs choose ‘same’ when presented with chemically identical stimuli (carbon chain length: *p* = 0.9564; functional group: *p* = 0.5881; Supplementary information Figures S4 and S5).

## Discussion

The aim of the current research was to ascertain whether dogs were able to learn a same-different concept with odour stimuli in order to determine whether this approach could then be applied to examine which odours dogs perceive as the same or different to each other. Our findings reveal that dogs can learn the same-different task with the trained odours, but they did not generalise this learning to novel stimuli. Indeed, learning the concept hinges on the ability to apply the rule to novel stimuli in order to rule out non-concept learning such as memorizing stimulus-specific rules. The fact that dogs can learn the same-different task is a promising finding in deepening our knowledge on olfactory perception in dogs, i.e. what do dogs perceive as same versus different. However, we do not know why dogs were not able to generalise the concept to testing stimuli. There are several possible factors which need to be considered in future research, but this was clearly a demanding task for dogs.

Our findings contrast with previous research examining odour matching on the basis of similarity (Marchal et al. 2016; Lazarowski et al. 2021); however, these tasks have some crucial differences. Matching to sample tasks, as used by Marchal et al. (2016) and Lazarowski et al. (2021), allow assessment of the stimulus which an animal perceives as most similar to a sample stimulus but do not allow us to determine whether the dog perceives them as the same, similar to that target or least dissimilar compared to the other choice stimuli (Johnen et al. 2017). Thus, the task itself may be less challenging for dogs to learn and/or it may be easier to generalise to novel stimuli as a result of this procedure. In the studies of Marchal et al. (2016) and Lazarowski et al. (2021) the dogs were correct in over 80% of their choices. However, because of the nature of the task, the use of this sort of approach does not allow assessment of their perception of odour space to be assessed except in relation to another stimulus.

In our study, dogs were able to learn the stimulus specific rule with the training stimulus set, but did not generalise this rule to novel stimuli, showing that they did not learn the abstract rule. It is unclear why they were unable to do this, however, there are several possible factors which should be considered. A potential explanation would be that the dogs had simply memorised the combinations in training rather than having learned the rule and there is evidence that animals can learn large numbers of stimuli (e.g. Fagot and Cook 2006), however, we did not see more rapid learning of the stimuli that were only presented as the same and only as different, making this less likely. It is possible that training the dogs further after acquisition of the task may have resulted in better generalisation of the rule to novel stimuli. Moreover, it is also possible that the complexity of the task may have imposed too great a cognitive load on the dogs (Sweller 2011). The learning of the same-different concept by understanding the link between the two stimuli presented simultaneously at each trial may have overloaded cognitive capacity resulting in a failure to generalize the task rules (Sweller 2011). Moreover, as noted in the methods, due to the apparent difficulty of the task, some of the dogs were only able to perform one block per day otherwise their performance dropped substantially. A similar finding has been reported by Smith and colleagues (2021), where they found that dogs were less likely to find an object as they proceeded through the trials within a block (where a block corresponds to 20 trials). The authors suggested that as the task progressed, the dogs were less attentive (Smith et al. 2021). Cognitive load may also have affected the dogs’ information processing and/or motivation to complete the task (Moko et al. 2023). In the current research, previous experience in olfactory detection work did not appear to help dogs in their performance as suggested by Caldicott et al. (2024). In fact, if anything, the opposite effect was observed; 3 out of the 4 dogs who met criteria in the training phase and participated in the testing phase had never taken part in a scent experiment before (Alfie, Fable and Freda). A potential explanation for this may be that dogs who had taken part in more traditional discrimination/generalisation odour experiments previously (4 out of the 10) were more likely to focus on the specific odours rather than the relationship between the two stimuli presented.

Finally, the challenging nature of the task may have induced a negative emotional state, such as frustration for some dogs. Previous research in dogs has found poor performance to be associated with negative emotional states (Haverbeke et al. 2008; Rooney et al. 2009; Arnott et al. 2014). Frustration can be expected to further inhibit their inability to learn the concept as learning becomes more difficult under strain and when animals perceive less control over reinforcement (Haverbeke et al. 2008; Rooney et al. 2009; Arnott et al. 2014). An increase in arousal during the task is also likely to have influenced their ability to generalize, as previous research has linked an increase in arousal with a decrease in cognitive performance in pet dogs (Bray et al. 2015), although each dog (like humans) is likely to have their own Individual Zone of Optimal Functioning (Ruiz et al. 2017). Research in humans has shown that an increase in arousal could favour habitual responding to the detriment of cognitive flexibility during a task (Schwabe et al. 2010).

The chemical properties of the stimuli used in testing may have made the generalisation of the concept extremely challenging to the dogs. Our knowledge of the mechanisms involved in the perception and classification of odours by dogs remains very limited. The training stimuli used were complex odour stimuli with which the dogs were more likely to have had previous experience. Conversely, the stimuli used during testing were monomolecular odour stimuli, giving them very distinctive odours and with which the dogs were unlikely to have had previous experience. Moreover, these testing stimuli were chemically different as they were alcohols and acids. Given our very limited knowledge on the mechanisms involved in the perception of odours by dogs, it is possible that the testing stimuli activated different olfactory receptors which resulted in different signals to the olfactory cortex compared to the training stimuli; perhaps the same phenomenon may apply between alcohols and acids. Indeed, this hypothesis is strengthened by the research of Uchida et al. (2000) which looked at glomeruli activity in the olfactory bulb of rats in order to understand their sensory map. They found that in the dorsal olfactory bulb, glomeruli in the lateral domain were activated by alcohols, ketones and some esters (e.g. β,γ-hexenyl acetate) whereas glomeruli in the anteromedial domain were activated by acids, aldehydes, and other esters. This finding can explain the results of DeGreeff et al. (2020) in which dogs trained on pentanoic acid can generalise to the related aldehyde and methyl ester but not to the alcohol or ketone. Therefore, it is possible that our choice of stimuli, which varied in terms of chemical properties and potentially activated different areas of the olfactory cortex, may have potentially affected the generalisation of same-different concept to the testing stimuli. Future research should therefore focus on training dogs with stimuli whose chemical properties are similar to those used in the tests.

In summary, we showed that dogs can learn a same-different task with odour stimuli, but did not generalise this rule to novel stimuli. Despite the absence of transfer, this area deserves further investigation. The task, as it was set up here, appears to be very cognitively demanding for dogs. Future research should assess other approaches particularly those which may reduce the cognitive load on the dogs. In addition, further attention should also be paid to the chemical properties of the stimuli used. Assessing odour space in dogs is an important step in improving performance of detection animals and the approach used here can inform future approaches to this question.

## Supplementary Information

Below is the link to the electronic supplementary material.

Supplementary Material 1

## Data Availability

The data that support the study findings is available on reasonable request from the corresponding author at awilkinson@lincoln.ac.uk.
