# Supplementary material for: Same-different learning of odour stimuli in dogs
Source: Anim Cogn. 2026 Jan 18;29(1):14. doi: 10.1007/s10071-025-02035-z (PMC12819428; doi:10.1007/s10071-025-02035-z)
Supplement: Supplementary file 1 — Supplementary Material 1 [file 10071_2025_2035_MOESM1_ESM.docx]

**Same-Different Learning of Odour Stimuli in Dogs**

Claire Ricci-Bonot, Amelia Duncan, Daniel S. Mills, Thomas W. Pike, Helen Zulch & Anna Wilkinson

Animal Behaviour, Cognition and Welfare Group, Dept of Life Sciences, University of Lincoln, Lincolnshire, LN6 7DL United Kingdom

Claire Ricci-Bonot: 0000-0002-6484-1904

Daniel S. Mills: 0000-0002-4765-9625

Thomas W. Pike: 0000-0002-6942-0498

Helen Zulch: 0000-0001-8933-6421

Anna Wilkinson: 0000-0002-4500-0181

Corresponding Author: Anna Wilkinson [awilkinson@lincoln.ac.uk](mailto:awilkinson@lincoln.ac.uk)

# Material and methods

## **Pre-training**

On arrival dogs were brought into the testing room where they were allowed to investigate off lead to become accustomed to the environment and the experimental set up. This habituation period to the testing room finished once the dog sat, lay down or returned to the experimenter in a relaxed or positively expectant manner. The maximum time allowable for habituation was set at 20 minutes. If the dogs did not habituate they were excluded from the study.

Once the dogs were habituated to the testing room, one of the experimenters trained the dog to:

1. perform two separate behaviours (e.g. sit, lie down, chin rest, paw on a box, go to a mat – the behaviours chosen depended on individual dog or owner preference (e.g. if the dog had musculoskeletal difficulties, then sit/lie down would have been avoided)) on separate verbal cues and sustain the behaviours for at least 2 seconds.
2. approach the stimulus presentation platform and sniff both pots on verbal cue (“go sniff”).
3. perform one of the two behavioural responses previously learnt on verbal cue (e.g. sit vs lie down) once they had sniffed both samples (as per Wright et al., 2017) depending on whether the odour samples were the same or different. For example, in the case where the two odour samples were identical, the experimenter said ‘sit’ to indicate to the dogs the behavioural response expected in this situation, on the other hand, if the two odour samples were different the experimenter said ‘lie down’.

To meet criteria to move onto the next phase of pre-training the dogs had to perform the correct behaviour following the verbal cue and hold it for 2 seconds in at least 24 trials in 3 blocks, with the last 5 trials correct (Wright et al., 2017).

## **Choice of stimuli and concentration of stimuli used during sample preparation**

*Table S1: Quantities of stimuli and mineral oil (for dilution) used in pre-training*

| Essential oil | Concentration | Essential oil (ml) | Mineral oil (ml) |
| --- | --- | --- | --- |
| Cinnamon | Low | 0.100 | 4.900 |
|  | High | 2.000 | 3.000 |
| Ginger | Low | 0.025 | 4.975 |
|  | High | 0.500 | 4.500 |
| Ylang-ylang | Low | 0.025 | 4.975 |
|  | High | 0.500 | 4.500 |
| Cedar | Low | 0.025 | 4.975 |
|  | High | 0.050 | 4.950 |

*Table S2: Complex (non-monomolecular) odour stimuli used in olfaction research in dogs and rats (*)*

| **Stimuli** | **References** |
| --- | --- |
| Antibiotic cream | Waggoner et al., 2022 |
| Coffee | Krichbaum et al., 2020 |
| Oregano | Krichbaum et al., 2020 |
| Paprika | Krichbaum et al., 2020 |
| Pine essence* | Keep et al., 2021 |
| Rosemary | Krichbaum et al., 2020 |
| Sesame (oil) | Krichbaum et al., 2020; Waggoner et al., 2022 |
| Liquid Soap* | Keep et al., 2021 |
| Tea (bag, tree oil) | Waggoner et al., 2022 |
| Vanilla | Krichbaum et al., 2020 |

*Table S3: Quantities of stimuli used during training and testing. Stimuli highlighted in yellow are liquid stimuli which were too viscous to be measured with a mechanical pipette so were instead weighed. Those highlighted in orange were measured using a mechanical pipette. Those without a highlight were weighed using a metal spatula and scales. Please note: both the high and low concentrations of the antibiotic cream were mixed with 2ml of distilled water to prevent the cream from sticking around the top of the glass vials, ensuring a consistent surface area between samples*

| Stimulus | High concentration | Low concentration |
| --- | --- | --- |
| Coffee | 0.3g | 0.1g |
| Tea | 0.4g | 0.1g |
| Rosemary | 0.5g | 0.2g |
| Oregano | 0.5g | 0.2g |
| Paprika | 1.5g | 0.5g |
| Liquid soap | 8g | 3g |
| Antibiotic cream | 3g | 0.5g |
| Sesame oil | 1ml | 0.1ml |
| Pine oil | 0.5ml | 0.05ml |
| Vanilla extract | 1ml | 0.05ml |

*Table S4: Quantities of carbon chain stimuli and mineral oil (for dilution) used in Testing*

| Stimulus | Molecule (ml) | Mineral oil (ml) |
| --- | --- | --- |
| Ethanol | 0.01 | 9.99 |
| Propanol | 0.01 | 9.99 |
| Butanol | 0.01 | 9.99 |
| Pentanol | 0.01 | 9.99 |
| Hexanol | 0.05 | 9.95 |
| Heptanol | 0.20 | 9.80 |
| Octanol | 0.50 | 9.50 |
| Pentanoic acid | 0.15 | 9.85 |
| 2-pentanone | 0.01 | 9.99 |
| 3-pentanone | 0.01 | 9.99 |
| Pentanal | 0.01 | 9.99 |
| Methyl valerate | 0.01 | 9.99 |
| Butyl butyrate | 0.05 | 9.95 |
| Ethyl cyanoacrylate | 0.10 | 9.90 |

*Table S5: Combinations of carbon chain stimuli used in Testing. Stimulus 1 and stimulus 2 could have been presented in either order, so their position in the stimulus presentation platform varied between dogs (e.g. one dog may have been presented with ethanol on the left and butanol on the right, while another dog had butanol on the left and ethanol on the right)*

| Stimulus 1 | Stimulus 2 |
| --- | --- |
| Ethanol (2C) | Ethanol (2C) |
| Propanol (3C) | Propanol (3C) |
| Butanol (4C) | Butanol (4C) |
| Pentanol (5C) | Pentanol (5C) |
| Hexanol (6C) | Hexanol (6C) |
| Heptanol (7C) | Heptanol (7C) |
| Octanol (8C) | Octanol (8C) |
| Pentanoic acid | Pentanoic acid |
| 2-pentanone | 2-pentanone |
| 3 - pentanone | 3 - pentanone |
| Pentanal | Pentanal |
| Methyl valerate (Methyl pentanoate) | Methyl valerate (Methyl pentanoate) |
| Ethanol (2C) | Butanol (4C) |
| Pentanol (5C) | Heptanol (7C) |
| Ethanol (2C) | Pentanol (5C) |
| Pentanol (5C) | Octanol (8C) |
| Propanol (3C) | Heptanol (7C) |
| Ethanol (2C) | Hexanol (6C) |
| Butanol (4C) | Octanol (8C) |
| Pentanol (5C) | Ethyl cyanocrylate |
| Pentanoic acid | 2-pentanone |
| Pentanal | 2-pentanone |
| Pentanoic acid | Pentanal |
| Methyl valerate (Methyl pentanoate) | 3 - pentanone |
| Pentanoic acid | 3 – pentanone |
| Pentanoic acid | Butyl butyrate |

# Results

## Training


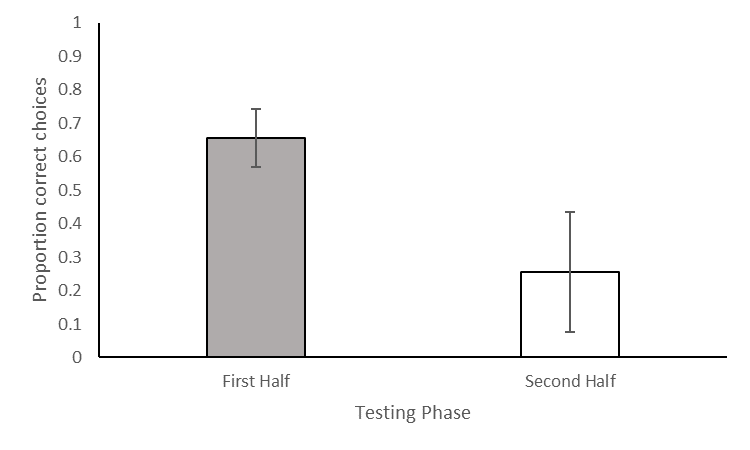


*Figure S1: Proportion of correct choices on the test trials in the first half of blocks compared to the second half of blocks for all dogs who partook in the test 1 phase (n=3 dogs)*

*Table S6: Number of blocks required to meet criteria for moving on to the testing phase. Re-training represents the number of blocks needed to return to criteria following the pilot test. Note: cells are highlighted in green when achieved and highlighted in light yellow at the stage of training when* *they have been withdrawn*

| **Subject** | **Training** | **Re-training** |
| --- | --- | --- |
| Wren | 24 | 15 |
| Fable | 14 | 11 |
| Alfie | 20 | n/a |
| Freda | 14 | n/a |
| Hector | 25 | 33 |
| Ghillie | 44 | n/a |
| Sol | 42 | n/a |
| Sumi | 42 | n/a |
| Talisker | 34 | n/a |
| Connie | 17 | n/a |


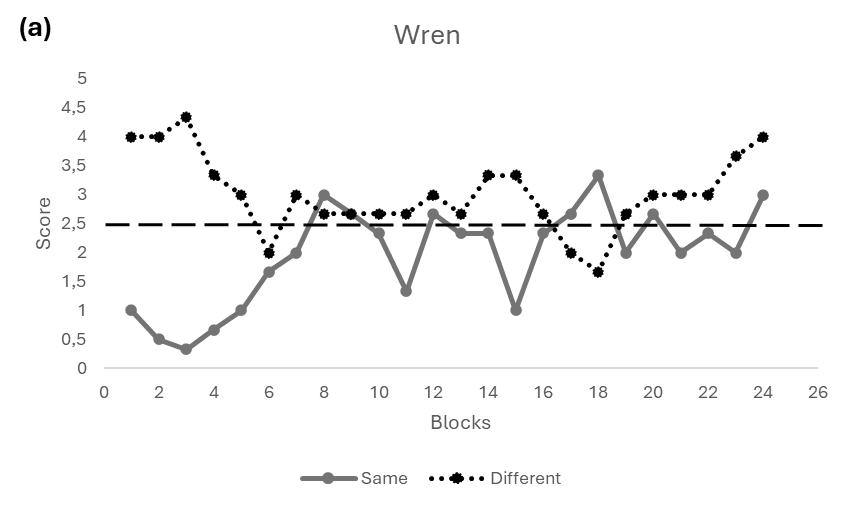

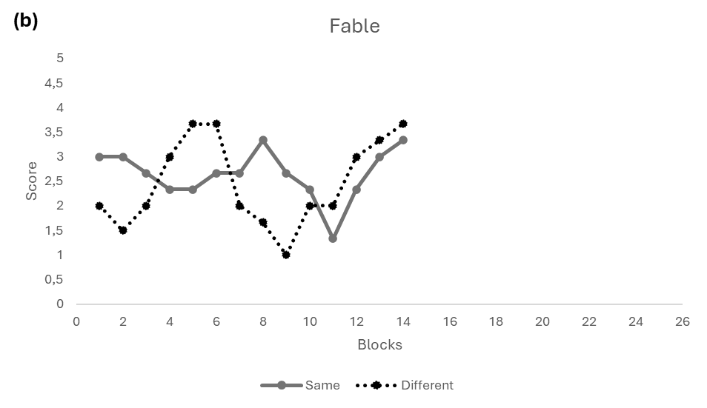


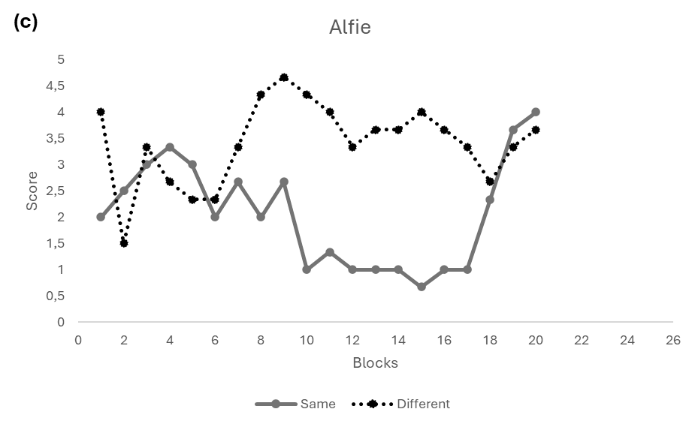

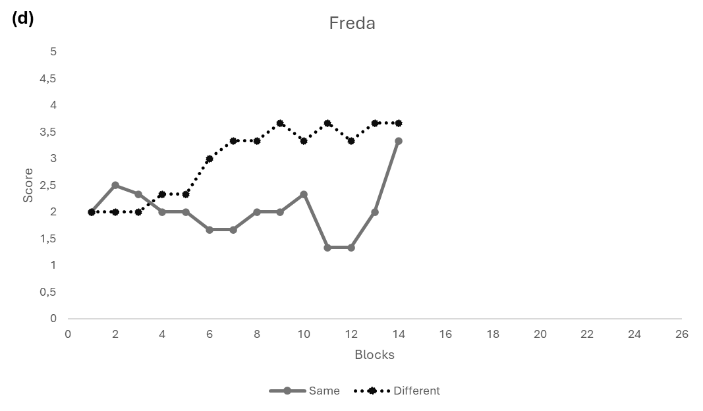


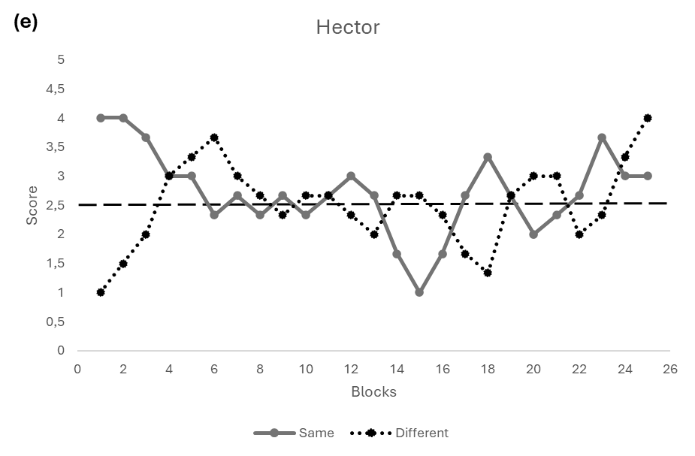


*Figure S2: Training graphs for all the dogs who met the criteria in the training phase, showing number of correct responses during same and different training trials in each block for Wren (a), Fable (b), Alfie (c), Freda (d) and Hector (e). Chance levels of performance are denoted by the dashed horizontal line.*


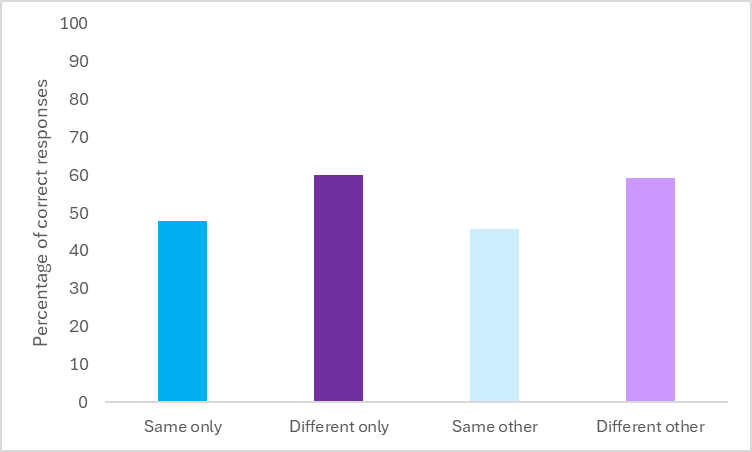


*Figure S3: Histograms for all the dogs who met the criteria in the training phase, showing percentage of correct responses during the training trials as a function of the stimulus pairings.*

## Testing

| All dogs (all tests) | Ethanol (2C) | Propanol (3C) | Butanol (4C) | Pentanol (5C) | Hexanol (6C) | Heptanol (7C) | Octanol (8C) |
| --- | --- | --- | --- | --- | --- | --- | --- |
| Ethanol (2C) | 2S + 2D |  |  |  |  |  |  |
| Propanol (3C) |  | 2S + 2D |  |  |  |  |  |
| Butanol (4C) | 2S + 2D |  | 3S + 1D |  |  |  |  |
| Pentanol (5C) | 2S + 2D |  |  | 1S + 3D |  |  |  |
| Hexanol (6C) | 1S + 3D |  |  |  | 4D |  |  |
| Heptanol (7C) |  | 3S + 1D |  | 1S + 3D |  | 1S + 3D |  |
| Octanol (8C) |  |  | 2S + 2D | 3S + 1D |  |  | 1S + 3D |
| Ethyl cyanocrylate |  |  |  | 2S + 2D |  |  |  |

*Figure S4: Perception as same or different of molecules which differ by carbon chain length (Note: Highlighted in black = dog(s) perceived them as different; highlighted in light grey = dog(s) perceived them as same; highlighted in dark grey = answers from multiple dogs which differ from each other. S denotes the number of ‘Same’ indication behaviours and D denotes the number of ‘Different’ indication behaviours displayed. The numbers indicate the number of dogs that encountered this combination, regardless of their performance in the intermixed training trials)*

| All dogs (all tests) | Pentanoic acid | 2-pentanone | 3 - pentanone | Pentanal | Methyl valerate |
| --- | --- | --- | --- | --- | --- |
| Pentanoic acid | 3S + 1D |  |  |  |  |
| 2-pentanone | 2S + 2D | 1S + 3D |  |  |  |
| 3 - pentanone | 2S + 2D |  | 1S + 3D |  |  |
| Pentanal | 3S + 1D | 3S + 1D |  | 4S |  |
| Methyl valerate |  |  | 2S + 2D |  | 1S + 3D |
| Butyl butyrate | 2S + 2D |  |  |  |  |

*Figure S5: Perception as same or different of molecules which differ by functional group (Note: Highlighted in black = dog(s) perceived them as different; highlighted in light grey = dog(s) perceived them as same; highlighted in dark grey = answers from multiple dogs which differ from each other. S denotes the number of ‘Same’ indication behaviours and D denotes the number of ‘Different’ indication behaviours displayed. The numbers indicate the number of dogs that encountered this combination, regardless of their performance in the intermixed training trials)*

## References

Keep B, Pike TW, Moszuti SA, Zulch HE, Ratcliffe VF, Porritt F, Hobbs E, Wilkinson A (2021) The impact of training method on odour learning and generalisation in detection animals. Appl Anim Behav Sci 236:105266. https://doi.org/10.1016/j.applanim.2021.105266

Krichbaum S, Rogers B, Cox E, Waggoner LP, Katz JS (2020) Odor span task in dogs (Canis familiaris). Anim Cogn 23:571-580. https://doi.org/10.1007/s10071-020-01362-7

Waggoner P, Lazarowski L, Hutchings B, Angle C, Porritt F (2022) Effects of learning an increasing number of odors on olfactory learning, memory and generalization in detection dogs. Appl Anim Behav Sci 247:105568. https://doi.org/10.1016/j.applanim.2022.105568
